# Engineering Cell Adhesion and Orientation via Ultrafast Laser Fabricated Microstructured Substrates

**DOI:** 10.3390/ijms19072053

**Published:** 2018-07-14

**Authors:** Eleftheria Babaliari, Paraskevi Kavatzikidou, Despoina Angelaki, Lefki Chaniotaki, Alexandra Manousaki, Alexandra Siakouli-Galanopoulou, Anthi Ranella, Emmanuel Stratakis

**Affiliations:** 1 Foundation for Research and Technology—Hellas (F.O.R.T.H.), Institute of Electronic Structure and Laser (I.E.S.L.), Vassilika Vouton, 711 10 Heraklion, Greece; ebabaliari@iesl.forth.gr (E.B.); ekavatzi@iesl.forth.gr (P.K.); angelaki@iesl.forth.gr (D.A.); manousa@iesl.forth.gr (A.M.); 2 Department of Materials Science and Technology, University of Crete, 70013 Crete, Greece; mst1095@edu.materials.uoc.gr; 3 Department of Physics, University of Crete, 70013 Crete, Greece; 4 Department of Biology, University of Crete, 70013 Crete, Greece; siakouli@biology.uoc.gr

**Keywords:** cell adhesion, cell orientation, Schwann cells, topography, laser fabrication, soft lithography, polymeric materials

## Abstract

Cell responses depend on the stimuli received by the surrounding extracellular environment, which provides the cues required for adhesion, orientation, proliferation, and differentiation at the micro and the nano scales. In this study, discontinuous microcones on silicon (Si) and continuous microgrooves on polyethylene terephthalate (PET) substrates were fabricated via ultrashort pulsed laser irradiation at various fluences, resulting in microstructures with different magnitudes of roughness and varying geometrical characteristics. The topographical models attained were specifically developed to imitate the guidance and alignment of Schwann cells for the oriented axonal regrowth that occurs in nerve regeneration. At the same time, positive replicas of the silicon microstructures were successfully reproduced via soft lithography on the biodegradable polymer poly(lactide-*co*-glycolide) (PLGA). The anisotropic continuous (PET) and discontinuous (PLGA replicas) microstructured polymeric substrates were assessed in terms of their influence on Schwann cell responses. It is shown that the micropatterned substrates enable control over cellular adhesion, proliferation, and orientation, and are thus useful to engineer cell alignment in vitro. This property is potentially useful in the fields of neural tissue engineering and for dynamic microenvironment systems that simulate in vivo conditions.

## 1. Introduction

Cell behavior in vivo is influenced by a variety of extracellular signals. It is currently clear that many cellular aspects, including adhesion, migration, spreading, proliferation, survival, apoptosis, and gene expression, are modulated by interdependent signaling cascades of soluble signals, shear stresses, other supportive cells, and the nature of the extracellular matrix (ECM) [1]. Thus, the main challenge in and goal of tissue engineering is to mimic the features of the ECM and the surrounding environment of cells sufficiently so that cells function in the artificial medium as they would in vivo [2]. Furthermore, individual cells recognize structures that have comparable dimensions to the those at the cellular level, which is at the micro scale. Consequently, control over micro/nanotopography is desirable. At the cell–material interface, all the cellular processes are governed by the physical and chemical stimuli of substrate stiffness (or rigidity), topography, and chemistry, respectively, while at the intracellular level, focal adhesions are key molecular complexes for sensing the environmental conditions as significant mechanosensitive players [3,4,5,6]. Indeed, many studies confirmed that the surface topography influences the adhesion, migration, polarization, proliferation, and differentiation of cells [7,8,9,10,11,12]. These parameters are of high significance for the design and development of advanced biomaterials in regenerative medicine and tissue engineering. Therefore, a considerable amount of research is devoted to the modification of materials’ surfaces for use as platforms to study cell viability, differentiation, motility, and apoptosis [13,14].

Generally, there are various materials and fabrication techniques that aim to reconstruct the ECM architecture in vitro with very specific compositions, ligand presentations, mechanical properties, and organization that vary between different tissues [13,14,15]. Indeed, previous studies have detailed the major fabrication techniques, the produced types of micro/nanostructured substrates, and advantages/disadvantages of the techniques [16,17]. Among various techniques that have been developed for surface modification, laser irradiation has proved to be important in the enhancement of material biocompatibility, particularly via the creation of new functional groups and the precise topography formation at the cellular and subcellular scale [10,18,19,20,21]. In particular, microstructuring via ultrafast lasers provides unique control over the uniformity and regularity of micron and submicron features [22].

The in vitro guiding of neurite outgrowth is important in tissue regeneration and for the development of neuronal interfaces with useful characteristics. To date, this has been achieved with micro- and nanofabrication techniques that give rise to various anisotropic continuous or discontinuous geometries [13,14]. Previous studies have demonstrated that anisotropic continuous electrospun polymeric fibers can influence neurite growth, alignment, and differentiation [23,24,25]. It has been also reported that in photolithographically fabricated continuous grooved substrates, axons grew on top of the ridges [25,26,27]. Moreover, studies have contributed significant insight into the impact of the disordered/anisotropic nanotopographical features on neuron differentiation and maturation by mechanotransduction pathways in PC12 cells [28,29,30]. It has also been reported that laser-microstructured discontinuous Si substrates not only support cellular adhesion and viability, but also significantly affect cell morphology, growth, orientation, and differentiation in a surface-dependent manner [10,20,21]. Furthermore, it was reported that both Schwann cells and axons of sympathetic neurons were parallel oriented on microcone patterns of elliptical cross-sections, while they exhibited a random orientation on the microcones exhibiting arbitrarily shaped cross-sections. As a result, it is suggested that an anisotropic continuous and discontinuous topographical patterns could promote Schwann cell and axonal alignment, provided that the pattern presents anisotropic geometrical features, even though their sizes are at a subcellular scale [20]. The same topographical model was used to study PC12 differentiation after treatment with nerve growth factor (NGF). It was shown that, unlike surfaces with low and medium roughness, those that are highly rough and exhibit large distances between microcones did not support PC12 cell differentiation, although cells had been stimulated with NGF [21]. Such substrates were also shown to support macrophage adherence and antigen presentation process in vitro, and to induce specific antibody production upon implantation in vivo [31].

Soft lithography is used to produce substrates with distinct surface topographies at the nano- and micrometer scale. It has been successfully used to transfer well-defined microsized patterns from silicon or stainless-steel masters to surfaces of soft biomaterials [32,33], allowing the replication of controlled microenvironments and in-depth study of the influence of surface properties on cell behavior [34].

As mentioned in our previous studies, we have thoroughly characterized discontinuous Si surfaces as cell substrates, and we have extensively investigated cell-specific responses of various neuronal cell types to these surfaces. In this study, we aim to demonstrate the reproducibility (or not) of Schwann cell behavior—focusing on growth, adhesion, and orientation—on laser-patterned polymeric microstructures, including those made from polyethylene terephthalate (PET) and poly(lactide-*co*-glycolide) (PLGA), compared with the Si substrates. PET has been widely used for cell culturing, surgical suture material, and prosthetic vascular grafts due to its biocompatibility and its excellent mechanical strength and resistance [35,36]. Moreover, PLGA is a biocompatible and biodegradable synthetic polymer that is used in various microfabrication techniques to create patterned substrates for various applications in tissue engineering and regenerative medicine [37,38,39,40]. In particular, microstructured substrates with different continuous microgroove (MG) and discontinuous microspike (MS) topographies were fabricated via either ultrafast laser direct writing of 2D planar PET substrates [41], or through soft lithography of PLGA replicas from microstructured Si substrates, respectively. The morphological, topographical, wetting, and optical properties of these substrates were investigated, and then their interactions with Schwann cells (SW10)—a murine glia cell line—in terms of adhesion, orientation, and proliferation were determined.

## 2. Results and Discussion

### 2.1. Scanning Electron Microscopy (SEM) Images of Laser-Microstructured Substrates on PET (PET-MG) and PLGA-MS (1:10) Replicas (from Laser-Microstructured Si Substrates with Three Different Laser Fluences)

**Figure 1:**
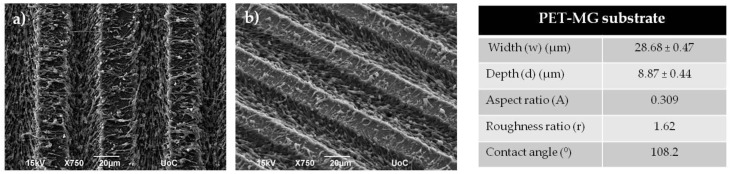
Scanning electron microscopy (SEM) images (top (**a**) and tilted (**b**) view) of polyethylene terephthalate microgroove (PET-MG) substrates. Measurements of the geometrical parameters of the surface of the PET-MG substrates in the tilted view of the SEM images were processed by Fiji ImageJ. A series of measurements were obtained for the surface characterization, such as width (w) and depth (d) of microgrooves, aspect ratio (A = d/w) and roughness ratio (r = 1 + 2d/w). Measurements of the contact angles were performed with the use of a tensiometer.

**Figure 2:**
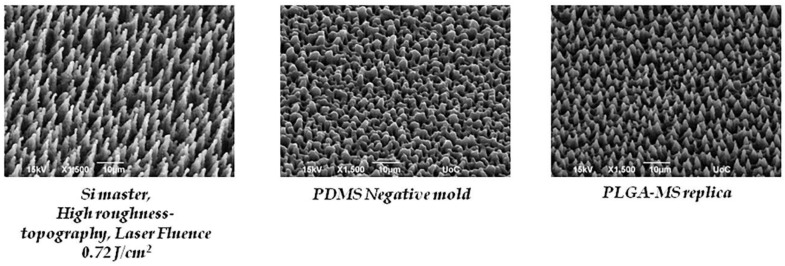
Scanning electron microscopy (SEM) images (tilted view) of a laser-microstructured Si-microspike (MS) substrate (high roughness/topography) mold, poly(dimethylsiloxane) (PDMS) negative mold, and poly(lactide-*co*-glycolide) (PLGA)-MS replica.

**Figure 3:**
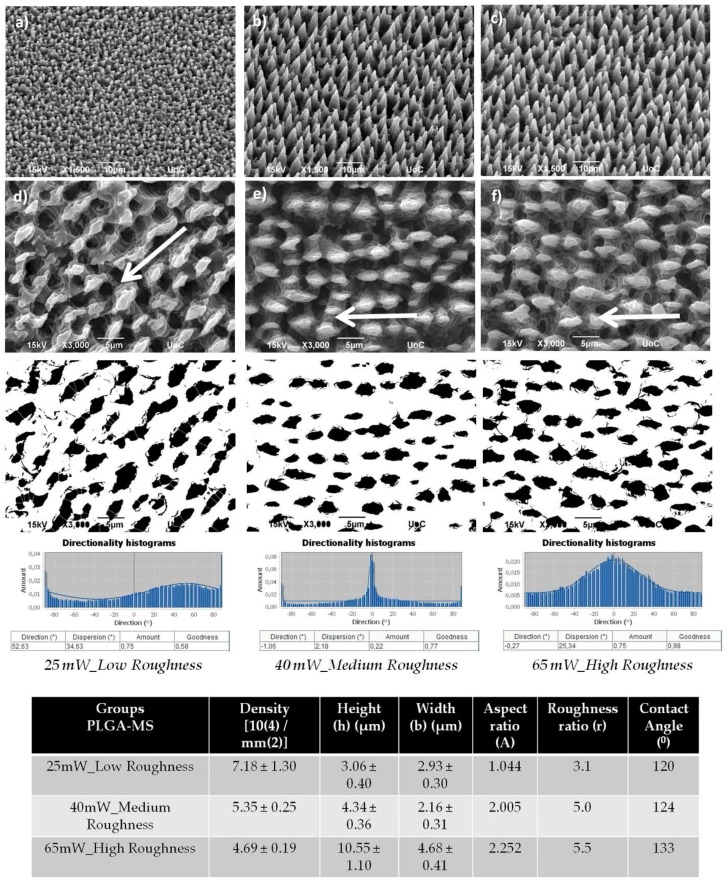
Scanning electron microscopy (SEM) images (tilted (**a**–**c**) and top (**d**–**f**) view) of PLGA-MS replicas of the three different topographies (25 mW_Low Roughness, 40 mW_Medium Roughness, and 65 mW_High Roughness). The white arrows represent the spikes’ direction. Under the images, directionality histograms and tables with statistics are presented, which were generated using the Fiji ImageJ plug-in “Directionality” [43]. Above the histogram, the plug-in generates statistics for the highest peak found. The highest peak is fitted by a Gaussian function, taking into account the periodic nature of the histogram. In the tables, the “Direction (°)” column reports the center of the Gaussian; the “Dispersion (°)” column reports the standard deviation of the Gaussian; the “Amount” column is the sum of the histogram from center-std to center+std, divided by the total sum of the histogram; the “Goodness” column reports the goodness of the fit, where 1 is good, 0 is bad. Measurements of the geometrical parameters of the surface of the PLGA-MS (10%) replicas at the three different topographies (25 mW_Low Roughness, 40 mW_Medium Roughness, and 65 mW_High Roughness) on the SEM images (top and tilted view) of the PLGA-MS replicas were processed using an image-processing algorithm (Fiji ImageJ) to determine the topological characteristics of the MSs. Measurements include the height (h), width (d), aspect ratio (A), and roughness ratio (r) from the top (highest magnification) and tilted-view SEM images. The aspect ratio was calculated by dividing the height by the radius of the spike’s base. For the PLGA-MS replicas, a surface plot of each image was produced by Fiji ImageJ, and the height and spike’s base were measured. From each image, at least 10 measurements were performed. The roughness ratio, r, was calculated by dividing the actual, unfolded, surface area of spikes by the total irradiated area (r = 1 + 2h/b, where b is the width of spikes). The mean value was calculated from four individual surfaces in each case. Measurements of the contact angles were performed with the use of a tensiometer.

Figure 1 depicts the SEM images of PET coverslips that were ablated by the femtosecond laser at a constant fluence of 11.9 J/cm^2^, scan velocity of 7 mm/s, and an x_step_ (distance between two consecutive scan lines) of 50 μm fabricated using a linear Gaussian beam. Thus, using these parameters, we fabricated microstructured substrates with a continuous microgroove geometry (PET-MG substrates). Such a surface morphology occurs due to the overlap between adjacent spots during the scanning process [42]. Figure 2 and Figure 3 respectively represent the different stages of the soft lithography process; from the Si master with microspikes, through the poly(dimethylsiloxane) (PDMS) negative mold, to the PLGA replicas with the three different topographies (Si-MS and PLGA-MS replicas). We have successfully reproduced (irradiated Si topography and PLGA replicas) patterned substrates exhibiting 2D–3D surface characteristics, resulting in an additional parameter to control cell growth and network formation. As shown in Figure 3, each culture substrate consisted of these three microstructured areas, irradiated using 0.42 J/cm^2^ (25 mW, low roughness), 0.58 J/cm^2^ (40 mW, medium roughness), and 0.72 J/cm^2^ (65 mW, high roughness). The main difference between the three PLGA-MS replicas is the distance between the spikes, that is, the spike size (aspect ratio). The higher the laser fluence, the higher the spike size, characterized as high roughness or topography.

The measurements of the geometrical parameters of the PET-MG substrates, as calculated from SEM images, are summarized in Figure 1. The width of the microgrooves was 28.68 ± 0.47 μm, the depth 8.87 ± 0.44 μm, the aspect ratio 0.309, and the roughness ratio 1.62. The geometrical characteristics of the spikes on the Si substrates have been previously determined [20,21]. Here, in Figure 3, we also show the measurements of the geometrical parameters of the surface of the PLGA-MS replicas for the three different topographies. As calculated from SEM images, the spike height varied from 3.06 ± 0.40 μm in the low-roughness structures to 10.55 ± 1.10 μm in the high-roughness structures (Figure 3). While spike density was the lowest in the high-roughness structures, the spikes’ height and roughness, thus aspect ratio, increased. These findings demonstrate the anisotropic nature of the PLGA-MS substrates. Furthermore, it is clear from Figure 3, and specifically from the directionality histograms, that there is a varied orientation between the replicas. The medium- and high-roughness PLGA-MS substrates showed a directionality at the area of zero degrees, while the low roughness substrate showed a lower directionality at the area close to 52 degrees.

### 2.2. Measurements of Wettability of Irradiated PET (PET-MG), Non-Irradiated PET (PET-Flat), and PLGA-MS (1:10) Replicas (from Irradiated Si Substrates)

The contact angle measured on the irradiated PET (PET-MG substrate) is presented in Figure 1. Specifically, the contact angle of the non-irradiated PET (PET-Flat) was ~77.8°, which is in agreement with previous studies [35,44], while the contact angle of the irradiated (PET-MG) is 108.2°. We observed a decrease in the hydrophilicity of the PET-MG substrate, which is attributed to the increased roughness of the surfaces after irradiation with the femtosecond laser [45]. Figure 3 shows the measured contact angles of PLGA-MS (1:10) replicas with the three topographies. Increasing the roughness of the PLGA-MS replica’s surface decreased the hydrophilicity. According to the literature, lactide is more hydrophobic than glycolide, therefore, PLGA copolymers rich in lactide (the PLGA in this study) are less hydrophilic and absorb less water, leading to a slower degradation of the polymer chains [46]. Therefore the topography enhances the degradation rate of this PLGA copolymer.

### 2.3. UV–Vis Measurements of Irradiated PET (PET-MG), Non-Irradiated PET (PET-Flat), and PLGA-MS (1:10) Replicas (from Irradiated Si Substrates)

**Figure 4:**
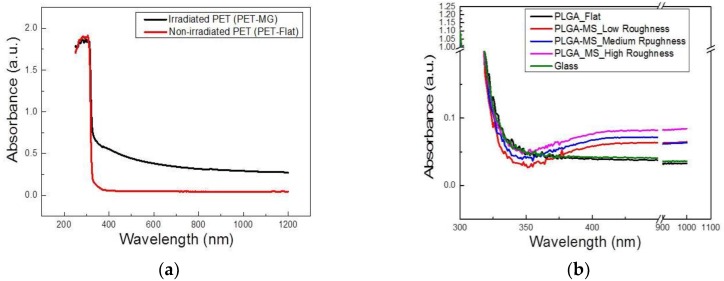
(**a**) UV–Vis measurements of irradiated PET (PET-MG) and non-irradiated PET (PET-Flat); (**b**) UV–Vis measurements of all the PLGA-MS replicas, as well as the PLGA flat and glass substrates.

In order to determine changes to the surface chemistry of the microstructured substrates, ultraviolet–visible (UV–Vis) spectroscopy was used. We observed an increase of the absorption in the irradiated PET (PET-MG) due to the structuring process. Moreover, we noticed the development of an absorption band in the region of 300–500 nm in PET-MG, likely due to the presence of aromatic hydroxylated species produced during the photooxidation of PET (Figure 4a), which is in agreement with previous studies [47,48,49]. Specifically, previous work [47,48,49] has demonstrated the development of an absorption band at around 340 nm in the UV range. The relevant absorbance (a.u.) exhibits an increase in the PLGA topographies compared with the flat PLGA and the glass substrate, as shown in Figure 4b. By increasing the topography or laser fluence of the PLGA-MS replicas, the absorbance (A) was increased (A_25 mW_Low Roughness < A_40 mW Medium Roughness < A_65 mW High Roughness). All absorption bands (obtained here by UV–Vis and unpublished data using ATR-FTIR on these replicas) found in the spectra agree with those given in the literature for PLGA copolymers [46,50]. There was a slight difference in the relevant absorbance of the microstructured replicas compared with the flat PLGA, but it was negligible.

### 2.4. Cell Seeding of Laser-Microstructured Substrates on PET (PET-MG) and PLGA-MS Replicas with Schwann Cells

**Figure 5:**
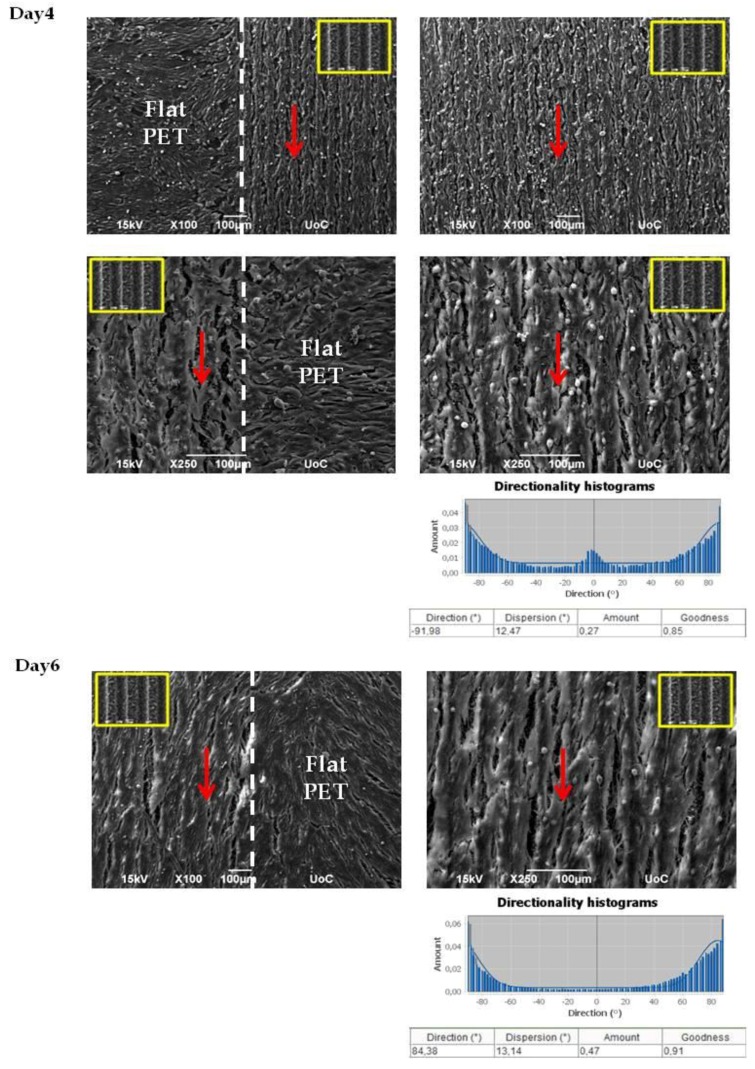
Scanning electron microscopy (SEM) images of Schwann cells cultured on the PET substrates (PET-MG and PET-Flat) for 4 and 6 days. The red arrows represent the directionality of Schwann cells, which are oriented according to the direction of the microgrooves. The inset SEM images, indicated by the yellow box, show the geometry of microgrooves. Under the SEM images, directionality histograms and the tables with statistics are presented, which were generated using the Fiji ImageJ plug-in “Directionality” [43]. Above the histogram, the plug-in generates statistics for the highest peak found. The highest peak is fitted by a Gaussian function, taking into account the periodic nature of the histogram. In the tables, the “Direction (°)” column reports the center of the Gaussian; the “Dispersion (°)” column reports the standard deviation of the Gaussian; the “Amount” column is the sum of the histogram from center-std to center+std, divided by the total sum of the histogram; the “Goodness” column reports the goodness of the fit, where 1 is good, 0 is bad.

In Figure 5, we show the morphology of Schwann cells for two different time points (4 days and 6 days) cultured on the PET substrates (PET-MG and PET-Flat). The anisotropic continuous microgrooves had a width of 28.68 ± 0.47 μm and a depth of 8.87 ± 0.44 μm. We noticed that the cells exhibited a branched shape and flattened morphology with long cellular extensions, which indicates good adhesion and growth of the cells on the microgrooves. Moreover, we noticed that the cells appeared to be oriented along the direction of the microgrooves for 4 and 6 days of culture, while they showed a random orientation on the flat PET. This is also demonstrated by the directionality histograms in Figure 5, which show that the amount is higher in the domain parallel to the microgrooves (±90 degrees). It is obvious from the SEM images in Figure 5 that, although surface roughness did not affect the proliferation of the cells (cells were equally grown on flat PET and PET-MG substrates), surface morphology significantly controlled the outgrowth of the cells. Consequently, cells could sense continuous directional topographical cues, with sizes at the subcellular scale.

**Figure 6:**
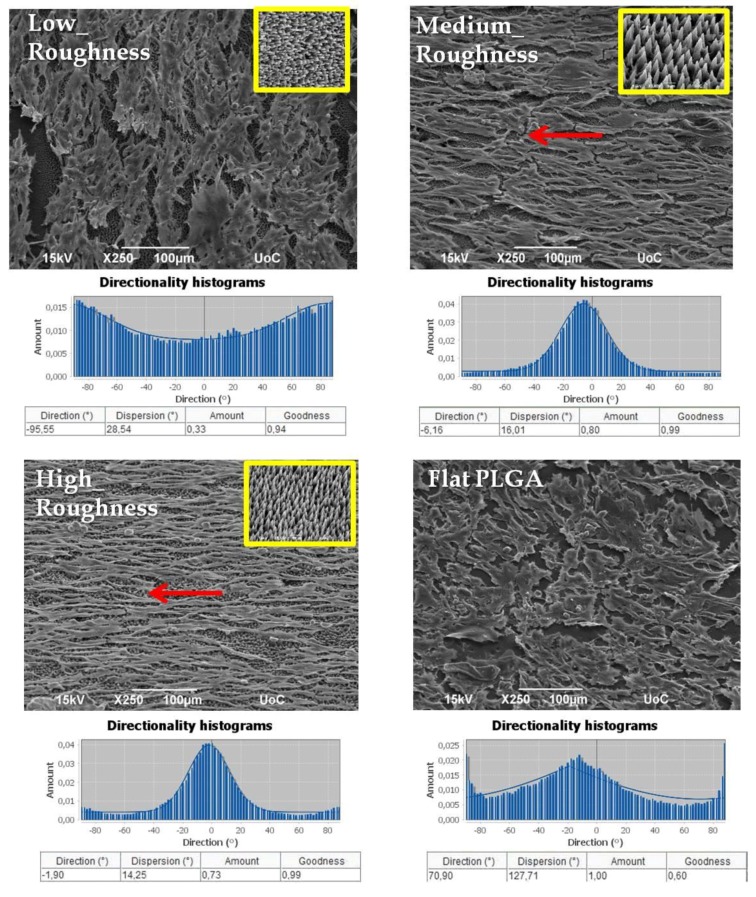
Scanning electron microscopy (SEM) images of Schwann cells cultured on the PLGA-MS replicas (three topographies) and on flat PLGA for 3 days. The red arrows represent the directionality of Schwann cells, which are oriented according to the topography of the PLGA-MS replica (inset SEM image, indicated by the yellow box, on the right side of each group). Under the SEM images, directionality histograms and tables with statistics are presented, which were generated using the Fiji ImageJ plug-in “Directionality” [43]. Above the histogram, the plug-in generates statistics for the highest peak found. The highest peak is fitted by a Gaussian function, taking into account the periodic nature of the histogram. In the tables, the “Direction (°)” column reports the center of the Gaussian; the “Dispersion (°)” column reports the standard deviation of the Gaussian; the “Amount” column is the sum of the histogram from center-std to center+std, divided by the total sum of the histogram; the “Goodness” column reports the goodness of the fit, where 1 is good, 0 is bad.

According to Figure 6, there is an apparent finding that all three discontinuous topographies on the PLGA-MS replicas equally support Schwann cells’ growth. It is demonstrated that a growth pattern/profile of the Schwann cells is observed mainly on the medium- and high-roughness PLGA-MS replicas, compared with the low-roughness PLGA-MS replica and flat PLGA substrate, for 3 days of culture. The cells adhered and aligned on the ridge of the spikes of surfaces with medium and high topography. On the contrary, on the flat substrate and on the low-roughness PLGA-MS replica, there is arbitrary cell growth occurring. Elongated cells are present in greater numbers on the high-roughness and the medium-roughness PLGA-MS replicas (as demonstrated from the directionality histograms in Figure 6, where there is a clear concentration of the quantity at the area of zero degrees) compared to low-roughness PLGA-MS replicas and flat PLGA. In this study, the topography (due to the laser irradiation process) of the polymeric substrates ranges at both the micro and nano scale. Our previous studies demonstrated that the adhesion, alignment, proliferation, and differentiation of different types of neural cells depend on the topography [12,13,14,15]. Specifically, it has been proved that there is directional cell outgrowth dictated by substrates of medium and high roughness [20]. In the present study, all the three topographies showed cell growth according to the spikes’ orientation (red arrows on Figure 6), indicating that the cells could sense the discontinuous directional topographical features at subcellular scales. To date, there are no widely accepted hypotheses regarding the mechanism for the effects of topography substrates on cell adhesion, orientation, and proliferation. Moreover, a study demonstrated that in microgrooved features, the ridge width is commonly larger than or equal to the size of a single cell, permissive for cell attachment and migration, as well as cell alignment following the geometrical guidance. In contrast, nanogrooved features are similar to the ECM architecture and are typically much smaller than a single cell, thus inducing cell alignment in a more fundamental way, such as mimicking or signaling the cell membrane receptors [51].

### 2.5. Fluorescent Images of Schwann Cells Seeded on Laser-Microstructured Substrates on PET (PET-MG) (Immunostaining) and PLGA-MS Replicas (Immunostaining)

**Figure 7:**
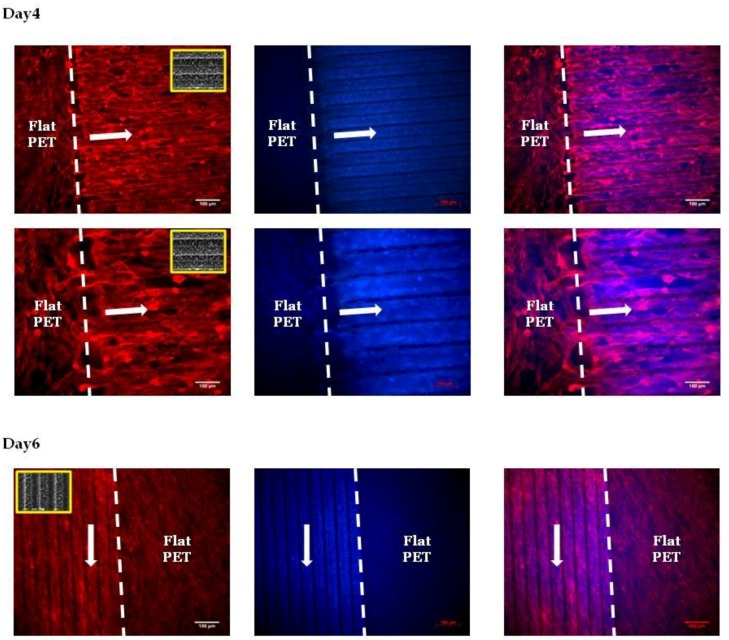
Fluorescent images of Schwann cells cultured on the PET substrates (PET-MG and PET-Flat) for 4 and 6 days. The cytoskeleton of the cells is visualized with red color (Alexa Fluor^®^ 568 Phalloidin), while the nuclei are indicated with blue color (DAPI). The white arrows represent the directionality of Schwann cytoskeleton, which is according to the direction of the microgrooves. The inset SEM images, indicated by the yellow box, show the geometry of microgrooves.

**Figure 8:**
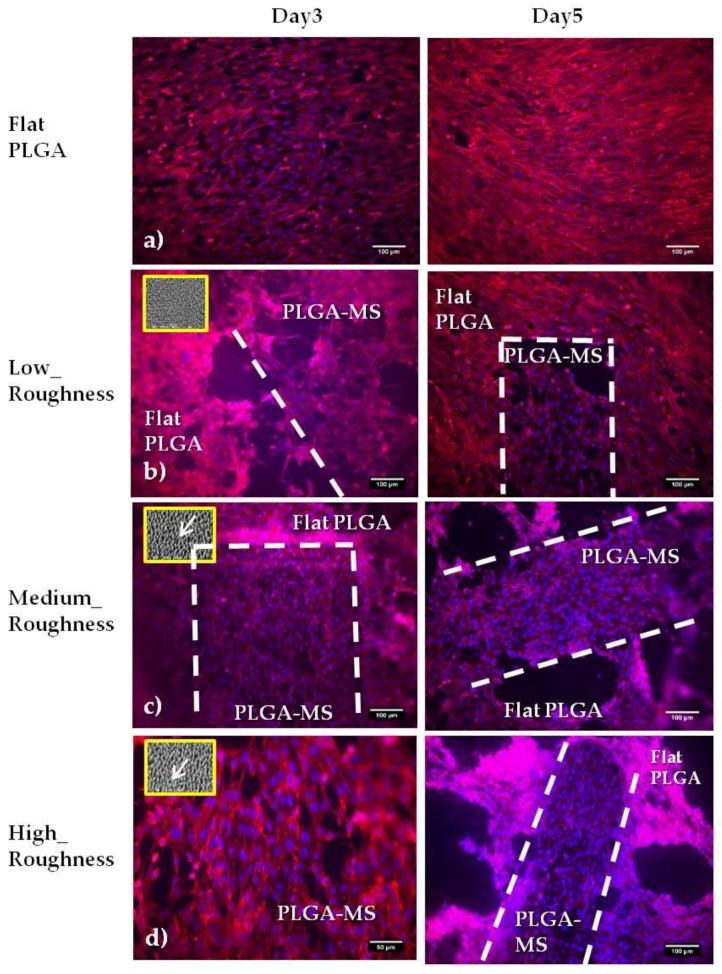
Fluorescent images of Schwann cells cultured on the (**a**) flat PLGA and PLGA-MS replicas (**b**–**d**) for 3 and 5 days. Each replica is defined by low (**b**), medium (**c**), and high (**d**) roughness. The cytoskeleton of the cells is visualized with red color (Alexa Fluor^®^ 568 Phalloidin). The white interrupted lines represent the PLGA-MS area, and thus the area with the spikes. It should be noted that for this specific study using the actin/DAPI assay, the PLGA-MS replicas (spike’s area) were 0.3–0.5 mm (width) and 1.5 mm (length). The inset SEM images on the left side, indicated by the yellow box, show the topography of the PLGA-MS replicas, and the white arrows represent the directionality of the spikes.

In Figure 7, we present the fluorescent images of Schwann cells cultured on the PET substrates (PET-MG and PET-Flat) for 4 and 6 days. The actin filament of cytoskeleton is visualized with red color, while the nuclei is indicated by blue color. We noticed that the cytoskeleton of the cells was elongated along the direction of the microgrooves, whereas a random orientation was observed on the flat PET. It is important to mention here that the width of the microgrooves is a critical parameter for the alignment of Schwann cells. The width of the Schwann cells ranges from 5 to 10 μm. It has been shown [14] that pattern widths or spacings varying from 2 to 30 μm are optimal for the alignment of Schwann cells. Indeed, when we used a topography of microgrooves with a width of 28.68 ± 0.47 μm, cells appeared to be oriented along the direction of microgrooves (as it is shown in Figure 7), while, when the width of the microgrooves was 168.12 ± 1.38 μm, a random orientation of cells was observed We found that PLGA-MS replicas seeded with Schwann cells resulted in the presence of elongated and round cells at the proliferation stage and signs of orientation according to the spikes (3 days of culture). Specifically, Schwann cells grew more randomly and in an isotropic manner on low-roughness PLGA-MS, comparable with the growth on flat PLGA. On medium- and high-roughness PLGA-MS replicas, the cells exhibited a directional growth. At the fifth day, the presence of elongated cells at different layers was observed, and there was full coverage of the surface. There was no difference between the three different topographies and the control substrate at this time point (Figure 8).

**Figure 9:**
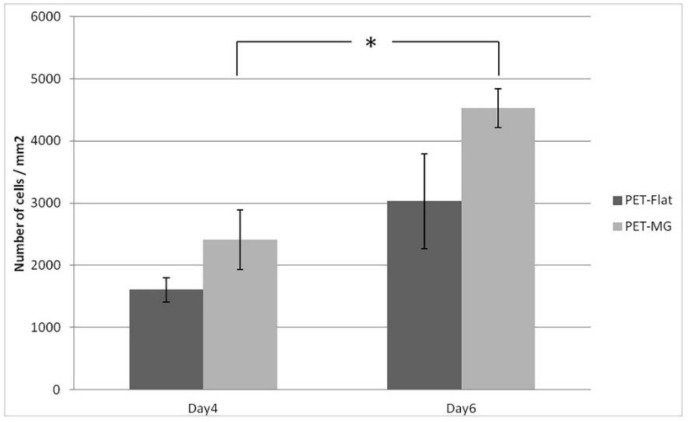
Proliferation of Schwann cells (number of cells/mm^2^) cultured on the PET substrates (PET-MG and PET-Flat) (via DAPI) for 4 and 6 days. The data were subjected to ANOVA with post hoc Tukey HSD test. A significant difference (* *p* < 0.0.5) was observed between 4 days and 6 days for the PET-MG substrate.

**Figure 10:**
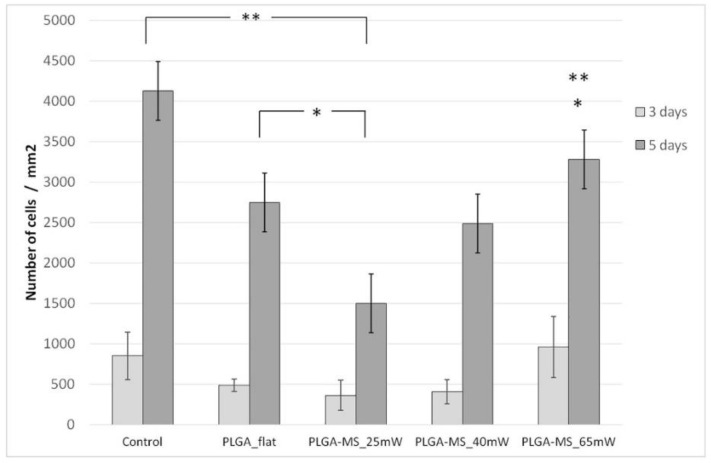
Proliferation of Schwann cells (number of cells /mm^2^) cultured on the PLGA-MS replicas, and PLGA flat and control samples (via live/dead assay) for 3 and 5 days. The data were subjected to ANOVA with post hoc Tukey HSD test for multiple comparisons between the groups. At 3 days, the *p* value > 0.05; therefore, the treatments (groups) were not significantly different for that level of significance. However, at 5 days, we observed some significant differences, strongly suggesting that one or more pairs of treatments (groups) are significantly different. In particular, the control group is significantly different from PLGA flat and PLGA-MS replicas of 25 mW_Low Roughness and 65 mW_High Roughness (** *p* < 0.001); PLGA-MS replica 25 mW_Low Roughness is significantly different from PLGA flat and the replica 65 mW-High Roughness (* *p* < 0.05).

The outgrowth of Schwann cells (number of cells/mm^2^) on the PET-MG substrate and on flat PET was evaluated by counting cell nuclei stained with DAPI (Figure 7). Nuclei number was assessed with Fiji ImageJ analysis. Figure 9 depicts the mean cell number on the PET-MG substrate and flat PET for 4 and 6 days of culture. The cell outgrowth was improved on PET-MG substrate compared to the flat PET, in agreement with the SEM and fluorescent images, with a significant difference between 4 days and 6 days at PET-MG. According to Figure 10, all three topographies of the PLGA-MS replicas support Schwann cells’ growth (results that are also confirmed from the SEM and fluorescent images) and proliferation for up to 5 days. The high-roughness PLGA-MS replicas had the highest cell number at both time points, followed by the medium-roughness PLGA-MS replica, while the lowest cell proliferation was observed for the low-roughness PLGA-MS replica. Taking into consideration Figure 8 and Figure 10, it is clear that these findings are in agreement with our previous work on Si microstructured substrates, where the surface roughness did not influence the Schwann cell growth, but the surface morphology (discontinuous pattern) played a key role in cell response [20]. Schwann cells seemed to be aligned with the orientation of the spikes’ topographical features and, specifically, this preference was more pronounced as the roughness increased. The key geometrical characteristics (height, width, and aspect ratio) of the substrates leading to the anisotropic nature of the spikes and their parallel orientation varied between the three topographies and significantly affected the degree of cell alignment. The previous findings of the group are also demonstrated in the present study, with cell growth on the low-roughness substrates having an isotropic manner similar to flat and control materials (shown clearly in Figure 8), and cell growth on medium- and high-roughness substrates exhibiting a more pronounced anisotropic growth (shown in Figure 6 and Figure 8). It should be noted here that the height of the spikes and the interspike distance cannot be controlled by the microfabrication techniques used in this study, and, since there is the step of replication of the topography (from the Si master mold, through the PDMS negative mold, to the final PLGA-MS replica), there is definitely a slight difference between the fabricated topography and the replicated topography in terms of the height of the spikes. These results demonstrate that the micro- and nanostructures favor the cell outgrowth.

In this study, we demonstrated that ultrafast pulsed laser irradiation is a simple and effective method to fabricate micro- and nanostructures with controlled geometry and pattern regularity. Two different synthetic polymers—the fabricated PET-MG substrates and the produced PLGA-MS replicas at a range of laser fluences, resulting in different levels of roughness, and geometrical characteristics were investigated for their selective cellular adhesion, proliferation, and orientation. In this context, we studied the effects of an anisotropic continuous topography and three anisotropic discontinuous topographies on cellular response.

**Figure 11:**
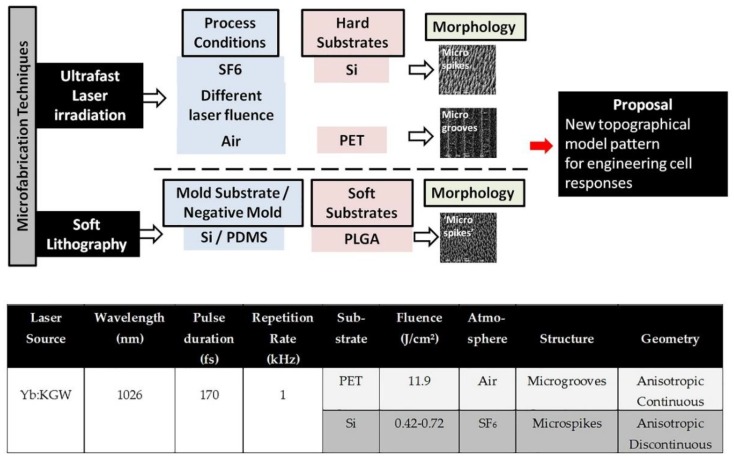
Comparison of the microfabricating techniques used in this study to fabricate the laser-microstructured substrates; the table demonstrates the conditions of the ultrafast laser irradiation process.

The morphological characterization of the PET-MG substrates and the PLGA-MS replicas (SEM images) indicated a topography with microgrooves (anisotropic continuous) for the PET substrates and microspikes (anisotropic discontinuous) for the PLGA replicas. This is due to the different fabrication processes used; PET substrates were laser-irradiated directly, and the PLGA-MS replicas were produced by soft lithography of laser-irradiated Si substrates. Thus, although the same laser irradiation process was used, the different materials formed a range of topographies, as shown in Figure 11. The composition and the mechanical properties of the material play a significant role in the topography [52]. The wetting and absorbance (related to optical properties) were assessed by the contact angle and the UV–Vis system, respectively. These properties were mainly affected by the topography of the material. Schwann cells attached strongly and proliferated on all the substrates. The cell adhesion/orientation engineering profile was mainly affected by the topography, while the cell proliferation was influenced by the topography.

The specific cell patterning model involving anisotropic continuous microgrooves (PET-MG) and anisotropic discontinuous microspikes with parallel orientations (PLGA-MS replicas) were developed in an attempt to imitate native nerve regeneration support structures, particularly imitating the guidance/alignment and growth of Schwann cells. It is known that primary Schwann cells transiently proliferate and form longitudinal bands of Bürger (boB) [53]. Aligned Schwann cells and their extracellular matrix are indispensable pathways for oriented axonal regrowth. The boB formation from a molecular point of view is unknown. A potential mechanism could be the polarized expression of adhesion proteins along the proximal–distal cell axis [53]. It was reported that placement of dissimilar adhesion characteristics in separate Schwann cell surface domains could aid longitudinal cell alignment. From a physical point of view, the basal lamina tube (enwrapping Schwann cells and myelinated axons) is the guiding cue for axonal regrowth [53].

Two different “axonal guidance”’ models were studied here. By using the same microfabrication techniques, two models were fabricated with different topographical (anisotropic continuous vs. discontinuous) geometries. The same cell type was tested. Schwann cells adhered, grew, equally aligned, and proliferated in both the models. Both models feature topographical cues (pattern) with a combination of nano- and microcharacteristics and are proposed to overcome the weaknesses of the existing and well-studied horizontal (grooves and ridges) or vertical (pillars, pores) cell patterning models.

The ability of this micropatterning strategy to control cellular adhesion and growth, and thus to engineer cell alignment in vitro, could be potentially useful in a wide range of neuroscience subfields, including basic research to understand cell interactions and network behavior; dynamic microenvironment systems that would better simulate the desired in vivo conditions; and, finally, neural tissue engineering, with the creation of implantable scaffolds for nerve tissue regeneration.

## 3. Materials and Methods

### 3.1. Experimental Setup Used for the Fabrication of Laser-Microstructured Substrates

**Figure 12:**
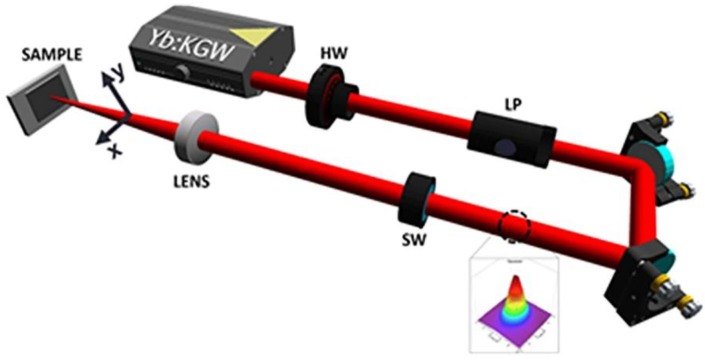
Experimental setup used for the fabrication of laser-microstructured substrates [42].

The microstructured substrates were prepared by ultrafast laser structuring, which is a simple but effective method to fabricate micro/nanostructures with different geometries [41]. The specially treated PET (polyethylene terephthalate) coverslips for cell culturing were subjected to laser irradiation. A Yb:KGW laser was used with a pulse duration equal to 170 fs, 1 kHz repetition rate, and 1026 nm wavelength. The beam propagated through a half waveplate and a linear polarizer (which were used to vary the values of power), to a shutter (that was used to control the exposure time and thus the number of pulses receptive to the sample), then to a convex lens of 10 mm focal length, and, finally, to the sample (Figure 12). The microstructured substrates were fabricated at a constant fluence of 11.9 J/cm^2^, scan velocity of 7 mm/s, and an x_step_ (distance between two consecutive scan lines) of 50 μm. The overall patterned area was 4 mm × 4 mm.

The same laser setup (Figure 12) was used to fabricate the Si substrates as described above. Single-crystal n-type silicon (1 0 0) wafers were subjected to laser irradiation in a vacuum chamber evacuated down to a residual pressure of 10^−2^ mbar. A constant sulfur hexafluoride (SF6) pressure of 650 mbar was maintained during the process through a precision microvalve system. A Yb:KGW laser was used with a pulse duration equal to 170 fs, 1 kHz repetition rate, and 1026 nm wavelength. The sample was mounted on a high-precision X–Y translation stage normal to the incident laser beam. The laser fluence used in these experiments was in the range 0.42–0.72 J/cm², thus creating three different topographies, defined as low, medium, and high topography [20,21]. The overall spike area was 5 mm × 5 mm. After laser irradiation, microstructured surfaces were morphologically characterized by scanning electron microscopy (SEM). The top SEM images (Figure 3d–f) revealed an arbitrarily shaped cross-section of the microstructures at low fluences that became almost elliptical as the laser fluence increased.

The laser-fabricated Si substrate is characterized as the “master” substrate. Negative replicas of the three master Si substrates were produced on elastomeric PDMS (SYLGARD 184, Dow Corning). In particular, liquid PDMS pre-polymer consisting of a “base” and “curing agent”, typically mixed in a 10:1 *w*:*w* ratio, was poured onto each substrate [54]. Then, the PDMS-coated Si substrates were placed into a vacuum chamber to remove residual air bubbles, thus providing for better penetration of the polymer into the laser microstructures. After heating at 80 °C for 2 h, a mold, which holds the negative of the original pattern, was peeled off of each Si substrate. An adequate number of PDMS negative molds was produced. Using the PDMS negative mold (negative spikes morphology), replicas of the initial morphology can be made out of several polymeric materials. In this study, we demonstrated the successful reproduction of the initial Si morphologies by producing PLGA replicas. A PLGA (lactide:glycolide 65:35, MW 40–75 k) polymeric solution of 1:10 (Code No: P2066, Sigma Aldrich, St. Louis, MO, USA) in dichloromethane (DCM) was carefully prepared. The PLGA solution was magnetically stirred for 2 h at room temperature (RT). One droplet of the PLGA solution was poured onto each PDMS negative mold and slowly finger-pressed with a glass disk. Following the evaporation of the solvent (24–48 h in −20 °C), the PLGA-coated PDMS mold was placed in 4 °C for 2 h. Then, the PLGA replica was peeled from the PDMS negative mold with a pair of tweezers.

### 3.2. Characterization of Laser-Microstructured Substrates

#### 3.2.1. Scanning Electron Microscopy (SEM)

The laser-microstructured substrates were morphologically characterized by scanning electron microscopy (SEM) (JEOL JSM-6390 LV, Jeol USA Inc, Peabody, MA, USA). Specifically, the substrates were sputter-coated with a 15 nm layer of gold (Baltec SCD 050, BAL-TEC AG, Balzers, Liechtenstein) and observed under the microscope with an acceleration voltage of 15 kV. Fiji ImageJ, an image processing software, was used to perform the analysis of the geometrical characteristics of the microgrooves and microspikes on the three topographies, as described in [20,21]. Briefly, the aspect ratio of the microgrooves/microspikes, A, was calculated by dividing the depth/height of the microgrooves/microspikes by the width. The roughness ratio, r, was calculated by dividing the actual, unfolded surface area of microgrooves/microspikes by the total irradiated area.

For the determination of the directionality of the PLGA-MS replicas, the “Local gradient orientation” for directionality was performed using the Fiji ImageJ plug-in “Directionality” [43].

#### 3.2.2. Wettability Measurements of Laser-Microstructured Substrates

The contact angles of the laser-microstructured substrates were calculated via an automated tensiometer, using the sessile drop method. A droplet of distilled, deionized Millipore water with a volume of 4 μL was positioned on the surface of the substrates using a microsyringe, and images were taken to measure the angle formed at the liquid–solid interface.

#### 3.2.3. UV–Vis Measurements of Laser-Microstructured Substrates

The UV–Vis absorption spectra of the laser-microstructured substrates were measured with a LAMBDA 950 UV/VIS/NIR spectrophotometer from Perkin Elmer with spectral range from 250 nm to 1200 nm. Laser-microstructured substrates and their relevant flat substrates were used for these measurements.

### 3.3. Cell Culture

The Schwann (SW10) mouse cell line is an established adherent neuronal Schwann cell line; it has been immortalized with SV40 large T antigen. SW10 cells were obtained from ATCC^®^ (Code: CRL-2766™). Schwann cells were grown in cell culture flasks using Dulbecco’s modified Eagle’s medium (DMEM (Invitrogen, Grand Island, NY, USA) supplemented with 10% fetal bovine serum (Biosera, Sussex, UK) in a 5% CO_2_ incubator (Thermo Scientific, OH, USA) at 33 °C. Laser-microstructured substrates were UV sterilized and transferred into sterile wells of 24-well plates (Sarstedt; Numbrecht, Germany). Then, 3 × 10^4^ cells in culture medium were seeded on the substrates and were cultured for a series of different time periods depending on the substrates, ranging from 3 to 6 days. The cell orientation and proliferation were better assessed at 4 and 6 days for the PET substrates, while for the PLGA replicas, the optimized time points were 3 and 5 days. The control samples in all the experiments were PET (polyethylene terephthalate) coverslips for cell culture.

#### 3.3.1. Morphology of Schwann (SW10) Cells by Scanning Electron Microscopy (SEM)

The laser-microstructured substrates seeded with the SW10 cells were removed from the incubator, washed twice with 0.1 M sodium cacodylate buffer (SCB), and fixed with 2% glutaraldehyde (GDA) and 2% paraformaldehyde (PFA) in 0.1 M SCB for 30 min. Thereafter, they were washed twice with 0.1 M SCB and dehydrated in increasing concentrations (from 30–100%) of ethanol. Finally, they were dried in a critical point drier (Baltec CPD 030, , BAL-TEC AG, Balzers, Liechtenstein), sputter-coated with a 15 nm layer of gold (Baltec SCD 050, BAL-TEC AG, Balzers, Liechtenstein), and observed under a scanning electron microscope (JEOL JSM-6390 LV, Jeol USA Inc, Peabody, MA, USA) at an accelerating voltage of 15 kV. For the PLGA replicas, CPD cannot be used since it deforms the polymer, so, after an optimization process, a hexamethyldisilizane (HDMS) protocol was established. After the dehydration steps with ethanol (EtOH), EtOH:HDMS solutions (50:50) were used for specific time points for all the replicas, and then the same procedure was repeated with HDMS solutions. Finally, the replicas were left to dry at room temperature overnight.

To investigate changes in the directional orientation of Schwann cells on the microstructured substrates, the “Local gradient orientation” for directionality was performed using the Fiji ImageJ plug-in “Directionality” [43].

#### 3.3.2. Immunocytochemical Assay

SW10 cells were stained for F-actin. Specifically, after 4 and 6 days of culture, the samples were fixed with 4% PFA for 15 min and permeabilized with 0.1% Triton X-100 in PBS for 5 min. The non-specific binding sites were blocked with 2% BSA in PBS for 30 min. Then, the samples were incubated for 2 h at room temperature with Alexa Fluor^®^ 568 Phalloidin (Invitrogen, Thermo Fisher Scientific) (1:250 in PBS–BSA 1%) for F-actin staining. Finally, the samples were washed with PBS and put on coverslips with DAPI (Molecular Probes by Life Technologies, Carlsbad, CA, USA) for nuclei staining. Cell imaging was performed using an epifluorescence microscope coupled to a high-resolution Carl Zeiss Axiocam color camera. The objectives of ×10 and ×20 were used. The number of SW10 cells that were grown on the microstructured substrates were determined by counting cell nuclei stained with DAPI. Nuclei number was assessed with ImageJ (cell counter plugin). The results represent the means of three different experiments (n = 10 field-of-view images for each substrate and time point).

#### 3.3.3. Live/Dead Assay

SW10 cells were seeded onto a PLGA replica to a density of 3 × 10^4^ cells/well. After 3 and 5 days of incubation under standardized culture conditions, medium was removed and replaced by a live/dead viability/cytotoxicity solution. The LIVE/DEAD™ Viability/Cytotoxicity Kit for mammalian cells (L3224, Thermo Scientific) was used for evaluating cell viability and proliferation. The cell-adhered replicas were washed twice with PBS. A live/dead solution was prepared by adding 20 μL of the supplied 2 mM ethidium homodimer-1 (EthD-1) stock solution to 10 mL of sterile PBS (thus reaching the desired concentration of 4 μM EthD-1 solution) and, after mixing thoroughly, 5 μL of the supplied 4 mM calcein AM stock solution was added to the 10 mL EthD-1 solution (thus reaching the desired concentration of 2 μM calcein AM solution). The solution was directly added to the replicas in order to cover the whole sample and was left for 45 min at room temperature. Finally, the cells were washed once with PBS, and fluorescent images were obtained by fluorescent microscope (images not shown here). The number of SW10 cells that were grown on the microstructured substrates were determined by counting cell nuclei stained with calcein. Nuclei number was assessed with ImageJ (cell counter plugin). The results represent the means of three different experiments (n = 10 field-of-view images for each substrate and time point).

### 3.4. Statistical Analysis

The data were subjected to ANOVA with post hoc Tukey HSD test to compare the significance levels (*p* < 0.05) between multiple groups.

## 4. Conclusions

Successful fabrication of micropatterned substrates was accomplished via ultrafast laser irradiation and soft lithography. Ultrafast pulsed laser irradiation is a simple and effective method to fabricate micro- and nanostructures with controlled geometry and pattern regularity. The anisotropic continuous (PET-MG) and discontinuous (PLGA-MS replicas) microstructured polymeric substrates were assessed in terms of their geometrical and topographical parameters (aspect ratio, roughness, and directionality), their influence on Schwann cell responses, and the reproducibility of these responses. The cells attached strongly and proliferated well on the substrates. Surface topography affected Schwann cells. Moreover, cells appeared to be oriented along the direction of the microgrooves and microspikes. This micropatterned strategy to control cellular adhesion and growth, thus engineering cell alignment in vitro, could be potentially useful in the field of neural tissue engineering and for the assessment in dynamic microenvironments by sufficiently simulating in vivo conditions.

## Abbreviations

2D	Two-dimensional
A	Absorbance
ATR-FTIR	Attenuated Total Reflection-Fourier Transform Infrared (spectroscopy)
BSA	Bovine Serum Albumen
CO_2_	Carbon dioxide
CPD	Critical Point Dryer
DAPI	4′,6-Diamidino-2-Phenylindole
DCM	Dichloromethane
DMEM	Dulbecco’s Modified Eagles Medium
ECM	Extracellular matrix
EthD-1	Ethidium Homodimer-1
EtOH	Ethanol
GDA	Glutaraldehyde
HDMS	Hexamethyldisilizane
IESL	Institute of Electronic Structure and Laser
MG	Microgrooves
MS	Microspikes
NGF	Nerve Growth Factor
PBS	Phosphate-buffered saline
PC12	Pheochromocytoma
PDMS	Poly(dimethylsiloxane)
PET	Polyethylene terephthalate
PFA	Paraformaldehyde
PLGA	Poly(lactide-co-glycolide)
RT	Room Temperature
SCB	Sodium Cacodylate buffer
SEM	Scanning Electron Microscopy
SF_6_	Sulfur hexafluoride
Si	Silicon
SW10	Schwann cells
UV	Ultraviolet
UV-Vis	Ultraviolet-Visible
UV/VIS/NIR	Ultraviolet/Visible/Near Infrared
Yb:KGW	Ytterbium-doped potassium gadolinium tungstate
